# Physician’s awareness of lung cancer screening and its related medical radiation exposure in Korea

**DOI:** 10.4178/epih.e2018002

**Published:** 2018-01-20

**Authors:** Seri Hong, Suyeon Kim, Mina Suh, Boyoung Park, Kui Son Choi, Jae Kwan Jun

**Affiliations:** 1 National Cancer Control Institute, National Cancer Center, Goyang, Korea; 2 Graduate School of Cancer Science and Policy, National Cancer Center, Goyang, Korea

**Keywords:** Lung neoplasms, Early detection of cancer, Radiography, Computed tomography, Radiation exposure, Health care surveys

## Abstract

**OBJECTIVES:**

Through a survey on perception of lung cancer screening and accompanying medical radiation exposure in Korea, the present study was to investigate its current situations and evaluate various perception of physicians regarding it in order to propose measures for improvements.

**METHODS:**

Medical specialists in national cancer screening institutions selected through stratified random sampling were subjected to face-to-face interview using a structured questionnaire. We investigated physicians’ perception on effectiveness of lung cancer screening depending on screening modality, selection criteria for subjects of screening, types of equipment used to screen, and perception for seriousness of adverse effects following the test. In addition, odds ratios to underestimate risk of radiation exposure from screening were calculated through logistic regression analysis.

**RESULTS:**

Each response that chest X-ray is effective for lung cancer screening and that smoking history is not considered prior to screening recommendation accounted for more than 60% of respondents, suggesting the chance of unnecessary screening tests. Regarding adverse effects of lung cancer screening, about 85% of respondents replied that false positive, radiation exposure, and overdiagnosis could be ignored. About 70% of respondents underestimated radiation dose from lung cancer screening, and a low proportion of physicians informed patients of radiation exposure risk.

**CONCLUSIONS:**

It was found that most physicians underestimated harms of lung cancer screening including radiation exposure and were lack of awareness regarding lung cancer screening. It should be noted that physicians need to have proper perceptions about screening recommendation and accompanying possible harms, for successful implementation of the screening program.

## INTRODUCTION

A clinical trial in the US, the National Lung Screening Trial (NLST), reported that regular screening of high-risk groups for lung cancer using low-dose computed tomography (LDCT) reduced lung cancer mortality [1]. Since then, many organizations have announced recommendations for lung cancer screening following the protocol of the NLST [2-6]. Various studies investigating physicians’ perception for the recommendations or current situations of lung cancer screening along with its inhibitory factors and concerns were conducted subsequently [7-13]. Those studies showed that the attitudes of physicians who recommend and order lung cancer screening test to eligible subjects should have great impacts on actual performance of lung cancer screening program. Accordingly, it was proven to be an important factor determines successful implementation of lung cancer screening system [14].

In Korea, recommendations on lung cancer screening using LDCT were newly announced in 2015 [15]. Currently, a pilot project has been ongoing to evaluate if it will be beneficial to introduce the screening protocol in the National Cancer Screening Program. Thus, it is first required that primary physicians and specialists in medical practices should have correct perception and attitudes to lung cancer screening in order to implement and settle a proper system. Since, in particular, LDCT involves 5-10 folds higher effective dose than other radiation tests such as chest X-ray and mammography [16,17], it is essential to consider radiation exposure in the assessment of benefits and harms of screening, which need to be properly evaluated and managed for introduction of the system.

Afterward, nevertheless, the guidelines for lung cancer screening in Korea was changed, there has been no report on actual situation of screening or survey on physicians’ perception in medical practices. Thus, in the present study, we conducted a survey on perception for lung cancer screening and accompanying medical radiation exposure with a sample population composed of specialists who were working in national cancer screening institutions in Korea, by which we evaluated what kind of modalities or protocols were used for actual screening, and physicians’ attitudes using a questionnaire. This study aimed to investigate current situations related to lung cancer screening in medical institutions, and to propose foundations for areas that require improvement or supplementation due to physicians’ lack of perception.

## MATERIALS AND METHODS

About 5,000 medical institutions that participated in the National Cancer Screening Program were targeted, of which 150 subject institutions were selected by stratified random sampling for each region and type of institution. Of these, we visited 104 institutions that consented through phone calls to participate in the survey, and conducted the survey on awareness of lung cancer screening through face-to-face interviews of one specialist per institution. The survey was conducted by a professional survey agency from January to February in 2013, in which a structured questionnaire was used to investigate perception about lung cancer screening and medical radiation exposure, along with the current situations of lung cancer screening and characteristics of individual respondents and their affiliated institutions.

In the survey on perception of lung cancer screening and medical radiation exposure, we investigated for awareness among all respondents about the effectiveness of lung cancer screening depending on screening method, their level of awareness about LDCT screening, and perceptions related to radiation exposure risk of lung cancer screening. Regarding awareness about the effectiveness of lung cancer screening, the question, “Do you think chest X-ray/LDCT screening is effective in reducing lung cancer mortality?” was asked depending on subject’s smoking history and screening modality. Measures of perceived radiation exposure risk of lung cancer screening were evaluated by the question, “What proportion of patients do you provide education about radiation exposure risk during lung cancer screening when using chest X-ray/LDCT?”

On the other hand, current situations of lung cancer screening were investigated only among respondents who replied ‘Yes’ to the question, “Do you or your affiliated institution conduct lung cancer screening?” They were asked if the decision to conduct screening depends on subject’s smoking history and age, and what kind of screening tests were performed during actual screenings. Through the question, “How serious do you think the potential adverse effects (false positive, false negative, radiation exposure, and overdiagnosis) of lung cancer screening are?”, perception about the harms of lung cancer screening and their seriousness were assessed.

Frequencies and percentages of each question were calculated and stated. Among the answers to five questions for evaluating perception of radiation exposure risk during lung cancer screening, “How risky do you think the total medical radiation dose by chest X-ray/LDCT is to health?”, “How serious do you think is the chance of adverse effects by radiation exposure during lung cancer screening?” and “How much do you estimate as an effective dose that subjects are exposed to during one chest X-ray/LDCT?”, each answer of ‘It is never risky’, ‘I have never considered it (ignore)’ and answers with lower radiation dose than that of chest X-ray or LDCT were counted. Based on those responses, the probability of underestimating radiation exposure risk from lung cancer screening was estimated by logistic regression analysis.

All survey results were collected and analyzed after completion of the survey period, in which personal information of respondents was kept confidential. The present study was approved by the institutional review board (IRB) of the National Cancer Center (IRB no. NCCNC2015-0095), and statistical analyses were performed using SAS version 9.4 (SAS Institute Inc., Cary, NC, USA) package.

## RESULTS

### Characteristics of respondents

Among the selected samples of 150 cancer screening institutions, a total of 104 institutions, or 104 specialists responded to the survey, resulting in about 69.3% of participation rate. Of these, 54 medical institutions (51.9%) answered that they were conducting lung cancer screening, and those respondents included 37 internal medicine specialists (68.5%), and 8 family medicine specialists (14.8%). In all, 44.2% of the total respondents had less than 20 years of experience after obtaining their medical practitioner’s license, and the remaining 55.8% had 20-40 years of experience. Their current affiliated medical institutions were mostly clinics (99 physicians, 95.2%), followed by hospitals (4 physicians, 3.8%) and general hospital (1 physician, 1.0%) (Table 1).

### Perception and current situations of modalities and validity of lung cancer screening

More respondents reported that both regular screenings using chest X-ray and LDCT were ‘effective’ (‘Very effective’ + ‘Somewhat effective’) in reducing lung cancer mortality than those who considered them ‘not effective’ (‘Never effective’ + ‘Little effective’). In addition, there were more responses that they were ‘effective’ for those with longer smoking history. About 17.3% of all respondents had an idea of the NLST study results from the US that reported the effectiveness of lung cancer screening using LDCT (Table 2).

Respondents who replied that they were conducting lung cancer screening were asked the question allowing multiple responses, “What kind of test is provided as lung cancer screening?” To this, 96.3% of them answered that only chest X-ray was used, whereas only 18.5% of the respondents were using LDCT. About 66.7% of the respondents did not consider smoking history when deciding to conduct screening, which was higher than the proportion of those who considered it. In contrast, a higher proportion of respondents (54.7%) considered age when deciding to conduct screening.

### Perception of hazards from lung cancer screening

**Figure 1.**
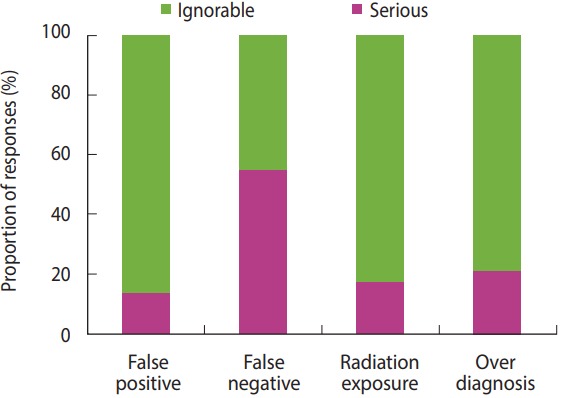
Physicians’ perceptions to the potential adverse effects of lung cancer screening (among the physicians who replied the institutions they belonged to were conducting lung cancer screening (n = 54); non-respondent (n = 1) was excluded).

**Figure 2.**
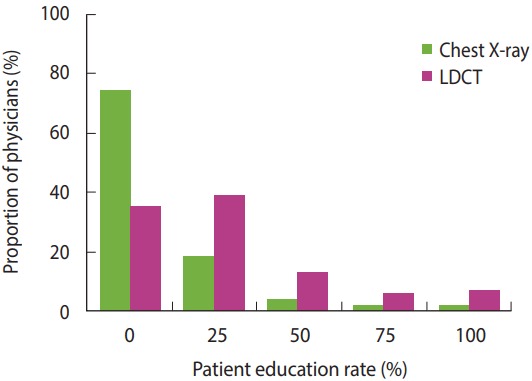
Proportion of providing patient education about radiation exposure before lung cancer screening via chest X-ray or LDCT (among the all respondents (n = 104); for LDCT screening, non-respondents (n = 4) were excluded). LDCT, low-dose computed tomography.

Among potential harms of lung cancer screening, the proportion of answer ‘It is serious’ (‘Very serious’ + ‘Serious’) recorded the highest (53.7%) for an adverse effect of ‘false negative’, whereas those proportions for ‘false positive’, ‘radiation exposure’, and ‘overdiagnosis’ were relatively lower with 13.0, 16.7 and 20.4%, respectively (Figure 1). When asked the proportion of patients who were provided with education about the radiation risk before chest X-ray or LDCT, the answer, ‘Absolutely none (0%)’ accounted for 74.0% for chest X-ray, corresponding about three quarters of all respondents; and 33.7% for LDCT, corresponding more than one third of all respondents (Figure 2).

### Perception of radiation exposure by lung cancer screening

Regarding hazards of total medical radiation dose by LDCT to the human body, 6.7% of the respondents replied ‘Never risky’. To the question for effective radiation dose of one LDCT imaging (among ‘0.009 mSv or less’, ‘0.01-0.9 mSv’, ‘1.0-4.9 mSv’, and ‘5.0- 9.9 mSv’), only 28.8% of the respondents selected the correct answer, ‘1.0-4.9 mSv’, whereas 67.3% selected lower doses. When asked about harmful effects of total radiation dose in chest X-ray on health and effective radiation dose of one shot chest X-ray (same answer choices with LDCT), 53.9% of the respondents chose ‘Never risky’, and 73.1% of the respondents selected lower doses than the actual dose (about 0.02 mSv). When asked if they were aware that adverse effects of radiation exposure during lung cancer screening could be serious, 5.6% of the physicians who answered that they conducted lung cancer screening, replied ‘Never considered (ignored)’. Based on the answers to the above five questions, we estimated the odds ratio (OR) of underestimating radiation exposure risk caused by lung cancer screening depending on characteristics of respondents and type of institution, which resulted in significantly lower ORs in specialists of internal medicine or family medicine than in other specialties (adjusted OR, 0.64; 95% confidence interval [CI], 0.43 to 0.96). Despite that it was not statistically significant, groups with women or those with less than 20 years in practice showed lower OR to underestimate radiation exposure risks than the rest (Table 3).

## DISCUSSION

Since the report of the US NLST study in 2011, protocols related with screening, prevention or care of lung cancer underwent many changes worldwide, which is still under discussion. The present survey study was performed with specialists in the institutions for national cancer screening after selection by stratified sampling, through which it was found to be an important prerequisite that physicians’ perception of lung cancer screening in practice need to be improved. The results indicated that there was a high proportion of physicians who had incorrect knowledge about benefits and harms of lung cancer screening tests. In particular, a considerable number of respondents underestimated risk of radiation exposure.

It has been continuously studied to find effective screening methods for lung cancer in the US and Europe since the mid-1900s. Early studies were mostly focused on chest X-ray, which finally reached to a conclusion that a frequent or regular chest X-ray was unable to decrease lung cancer mortality effectively, regardless of high-risk group [18]. Thus, most recommendations including the guidelines of Korea have emphasized not to conduct lung cancer screening using chest X-ray [3,4,15]. However, the present study found that a number of physicians in Korea have still perceived chest X-ray as an effective screening modality to reduce lung cancer mortality, and most of medical institutions have used chest X-ray for lung cancer screening. These raised important issues that an ineffective test caused unnecessary costs and radiation exposure.

On the other hand, most studies on lung cancer screening using LDCT, which started around 2000, found that LDCT had excellent outcomes in terms of early detection of lung cancer. In NLST study that is considered as a clinical trial with the highest level of evidence, LDCT was reported to indeed reduce lung cancer mortality [5,18]. Nevertheless, LDCT can be extremely limitedly used for screening due to uncertainty of accompanying harms. Mainly claimed harms include a high positive rate, its resulting unnecessary additional tests or interventional procedure, risk for its accompanying complications, radiation exposure, and overdiagnosis [3-5,15]. Although it is unlikely to have a serious harm, it does not mean that those adverse effects can be ignored. Particularly, it needs to be studied more on hazards of radiation exposure caused by regular screening, and it is difficult to conclude currently if such radiation exposure or risk from additional tests could be simply traded off for expected benefits of early detecting cancer or reducing mortality risk [5,19]. Therefore, experts emphasize that there should be proper protocols for additional tests after determined positive result or detection of solitary pulmonary nodule through lung cancer screening using LDCT, and that it is necessary to prepare a standard to minimize and control the exposure of radiation during screening [4,5,20].

In 2004, Brenner [21] studied adult smokers in 50-75 years of age who were screened using LDCT every year, and then reported excess risk of lung cancer caused by radiation exposure and a predicted number of additional patients with lung cancer by application of this risk to the entire subjects in the US. In a paper published 2014, McCunney & Li [22] reported cumulative radiation dose for 20 or 30 years when solitary pulmonary nodule, found by screening LDCT, was followed up using 8.0 mSv/session of chest CT according to the protocol of NLST study. In a paper in the Journal of the American Medical Association on benefits and harms of lung cancer screening using LDCT, cancer mortality by radiation exposure in NLST study was predicted using the radiation dose model from studies on survivors of nuclear bombing or medical radiation [5]. Although radiation exposure from screening could be tolerated due to its benefits, these studies suggested that it is necessary to perceive that radiation exposure should be an important issue to be considered in terms of public health if it becomes a population exposure, which means ‘screening program’, beyond individual level [16,22]. On the other hand, since the scale of individual hazards by radiation exposure can greatly vary depending on subjects’ age or gender, smoking history, degree of obesity, radiation exposure history, screening cycle or protocol [20,23], decisions whether and whom to recommend should be made considering those factors. Physicians’ perception needs to be improved through publication and distribution of proper screening guidelines, which reflects such considerations. Particularly, smoking and radiation exposure seem to have a potential synergistic effect, therefore smoking should be paid more attention [16,22].

The present study estimated risks to underestimate or ignore hazards of radiation exposure depending on characteristics of respondents, resulting in statistically significant difference depending on specialty. Compared to other specialties, those risks were significantly lower in specialists of internal medicine or family medicine, which showed that appropriateness and degree of understanding about screening could be different depending on specialty within mainly primary physician groups. In other words, when being trained with broader concept of medicine including primary and secondary preventions or screening during residency in internal medicine or family medicine, those physicians could correctly perceive accompanying harms or adverse effects of screening test. However, those who did not be trained with such concepts in other specialties should be supplemented for those lacking areas with continuous evaluation and management through residency or post-residency education. On the other hand, a number of respondents highly evaluated seriousness of false negative in lung cancer screening, which seemed to be affected by the fact that most of them considered chest X-ray as a screening method. In contrast, the reply that false positive, radiation exposure and overdiagnosis, which are major adverse effects of screening using LDCT, can be ignored, was as high as about 80.0%, showing a low perception of harms from screening. In addition, a number of respondents underestimated radiation dose caused by LDCT imaging. While it was reported that the mean effective radiation dose of one imaging with LDCT would be about 1.5-2.0 mSv [5,22,24], about 67.4% of the all respondents underestimated it. Moreover, radiation dose of CT can be in a wide range depending on type of equipment and its setting, and repeated annual screening followed by diagnostic tests, if needed, could further increase total effective dose, so that it is further required for physicians to have accurate understanding and perception.

As such, it is essential to be fully aware of screening recommendation, enabling to conduct appropriate screening for subjects using LDCT, which is regarded as the only effective screening modality, and minimize harms caused by unnecessary screening. In terms of the advantage of the present study, it evaluated healthcare providers’ perception of lung cancer screening and its current situations through the survey, by which it identified areas to be improved before introduction of a program and provided foundation to propose a measure to improve. However, the survey period was January–February 2013, which corresponded to an early time frame compared to 2012–2015 when major recommendations were announced in countries other than Korea and was much earlier than 2015 when Korean guidelines were announced, so this study is limited because overall perception about lung cancer screening using LDCT had to be relatively lower. If a same study were to be conducted as of now, the perception would be improved in many aspects. In addition, this study also had limitations in distribution and characteristics of medical institutions. Since most of participated screening institutions were clinics, the primary care unit, it was difficult to identify current situations of hospitals or general hospitals. Moreover, only a small proportion of the institutions, which replied that they conducted lung cancer screening, had LDCT equipment for screening. Accordingly, the proportion of lung cancer screening using LDCT was low, and it was highly likely that the answer to the question about “method for lung cancer screening” could be affected by the limited availability of LDCT in screening institutions rather than decision of respondents. Thus, it should be further investigated on current situations related with recent lung cancer screening, targeting more various medical institutions, and then it should be studied on measures to introduce and implement a system adapted and customized to the situation of Korea.

Our study is the first evaluation report of Korean physicians’ perception about lung cancer screening, which found that most physicians tended to underestimate harms of lung cancer screening including radiation exposure and had inappropriate perception about effective screening method and target subjects. Since benefits of screening that is conducted within both individuals and population should be considered in parallel with harms, each healthcare provider should correctly understand benefits and harms of lung cancer screening using LDCT and needs to determine based on accurate perception of guidelines when making decision to recommend screening or when selecting a screening method. In the future, it should be continuously discussed on balance between benefits and harms depending on the range of screening subjects, insurance coverage and cost-effectiveness, control of proper quality and establishment of its accompanying protocol. In addition, it is also required to enhance education of primary physicians and specialists, and suggestion of proper guidelines for decision-making procedure between physician and patient.

## Figures and Tables

**Table 1.** Characteristics of study subjects

Characteristics	Total	Physicians of self-reported lung cancer screening institutions^1^
No. of the respondents	104 (100.0)	54 (51.9)
Gender		
Man	83 (79.8)	47 (87.0)
Woman	21 (20.2)	7 (13.0)
Age (yr)		
30-39	15 (14.4)	7 (13.0)
40-49	49 (47.1)	24 (44.4)
≥50	40 (38.5)	23 (42.6)
Specialty		
Internal medicine	54 (51.9)	37 (68.5)
Family medicine	12 (11.5)	8 (14.8)
Others	38 (36.5)	9 (16.7)
Years after medical license issuance (yr)		
<10	8 (7.7)	9 (9.3)
10-19	38 (36.5)	5 (37.0)
20-29	49 (47.1)	11 (46.3)
≥30	9 (8.7)	15 (7.4)
Hospital type		
Clinic	99 (95.2)	51 (94.4)
Hospital	4 (3.8)	2 (3.7)
General hospital	1 (1.0)	1 (1.9)
Underestimation of the radiation exposure risk from lung cancer screening^2^		
Total number of responses	469 (100.0)^3^	269 (57.4)^4^
Underestimate	212 (45.2)	102 (37.9)
No underestimate	257 (54.8)	167 (62.1)

Values are presented as number (%). LDCT, low-dose computed tomography.

1 Physicians who replied the institutions they belonged to were conducting lung cancer screening.

2 Composed of 5 questions and corresponding answers, from which following responses for each question were defined as ‘underestimation’: (1) Answered ‘Never risky’ to the statement of “How risky do you think the total medical radiation dose by chest X-ray is to health?”; (2) Answered ‘Never risky’ to the statement of “How risky do you think the total medical radiation dose by chest LDCT is to health?”; (3) Answered ‘Never considered (ignored)’ to the statement of “How serious do you think is the chance of adverse effects by radiation exposure during lung cancer screening?”, among the physicians of self-reported lung cancer screening institutions (n=54) and non-respondent (n=1) was excluded; (4) Answered ‘<0.01 mSv’ to the question asking “Estimated effective radiation dose of one shot chest X-ray examination”; (5) Answered ‘<0.01 mSv’ or ‘0.01-0.9 mSv’ to the question asking “Estimated effective radiation dose of one LDCT imaging”.

3 The number of respondents was 104 (physicians) for 4 questions and 53 (physicians) for the other 1 question.

4 The number of respondents was 54 (physicians) for 4 questions and 53 (physicians) for the other 1 question.

**Table 2.** Physicians’ perceptions and practices about lung cancer screening

Questions	Yes	No
Among all respondents (n=104)		
Do you think chest X-ray screening is effective to lung cancer mortality reduction?		
In non-smokers	62 (59.6)	42 (40.4)
In past smokers	73 (70.2)	31 (29.8)
In current smokers	76 (73.1)	28 (26.9)
Do you think chest LDCT screening is effective to lung cancer mortality reduction?		
In non-smokers	59 (56.7)	45 (43.3)
In past smokers	86 (82.7)	18 (17.3)
In current smokers	91 (87.5)	13 (12.5)
Are you familiar with the results of the NLST study regarding LDCT lung cancer screening?	18 (17.3)	86 (82.7)
Among the respondents of self-reported lung cancer screening institutions (n=54)^1^		
Which screening tests do you order?		
Chest X-ray	52 (96.3)	-
LDCT	10 (18.5)	-
Sputum cytology	6 (11.1)	-
Do you recommend lung cancer screening tests depending on smoking status?	18 (33.3)	36 (66.7)
Do you recommend lung cancer screening tests depending on age?^2^	29 (54.7)	24 (45.3)

Values are presented as number (%). LDCT, low-dose computed tomography; NLST, National Lung Screening Trial.

1 Respondents who replied the institutions they belonged to were conducting lung cancer screening.

2 Non-respondent (n=1) was excluded.

**Table 3.** ORs for underestimating the risk of radiation exposure of lung cancer screening

Characteristics	Total no. of responses (5 questions)^1^	Underestimate^2^	No underestimate	Crude OR (95% CI)	Adjusted OR (95% CI)
Gender					
Man	379	169 (44.6)	210 (55.4)	1.00 (reference)	1.00 (reference)
Woman	90	43 (47.8)	47 (52.2)	1.14 (0.72, 1.80)	0.98 (0.60, 1.60)
Years in practice (yr)					
<20	209	91 (43.5)	118 (56.5)	0.89 (0.61, 1.28)	0.89 (0.62, 1.29)
≥20	260	121 (46.5)	139 (53.5)	1.00 (reference)	1.00 (reference)
Specialty					
Internal or family medicine	309	128 (41.4)	181 (58.6)	0.64 (0.44, 0.94)	0.64 (0.43, 0.96)
Others	160	84 (52.5)	76 (47.5)	1.00 (reference)	1.00 (reference)
Type of medical facility					
Clinic	446	201 (45.1)	245 (54.9)	0.90 (0.39, 2.07)	0.83 (0.38, 2.07)
Hospital	23	11 (47.8)	12 (52.2)	1.00 (reference)	1.00 (reference)

Values are presented as number or number (%). OR, odds ratio; CI, confidence interval.

1 The number of respondents was 104 (physicians) for 4 questions and 53 (physicians) for the other 1 question.

2 Criteria for ‘underestimation’ in each five question are described in manuscript (materials and methods section) and the footnote (number 2) of Table 1.

## SUPPLEMENTARY MATERIALS

Supplementary Material: Korean version is available at http://www.e-epih.org/.
