# Supplementary material for: Physician’s awareness of lung cancer screening and its related medical radiation exposure in Korea
Source: Epidemiol Health. 2018 Jan 20;40:e2018002. doi: 10.4178/epih.e2018002 (PMC6060336; doi:10.4178/epih.e2018002)

Volume: 40, Article ID: e2018002

https://doi.org/10.4178/epih.e2018002

Original Article

폐암 검진 및 관련된 의료방사선 노출에 대한 의료진 인식 조사

Seri Hong, MD, PhD[1] [orcid.org/0000-0002-2536-0606]
Suyeon Kim, MPH[2] [orcid.org/0000-0001-8922-2280]
Mina Suh, MD, PhD[1] [orcid.org/0000-0001-8101-7493]
Boyoung Park, MD, PhD[1,2] [orcid.org/0000-0003-1902-3184]
Kui Son Choi, PhD[1,2] [orcid.org/0000-0001-5336-3874]
Jae Kwan Jun, MD, PhD[1,2] [orcid.org/0000-0003-1647-0675]

[1]National Cancer Control Institute, National Cancer Center, Goyang, Korea
[2]Graduate School of Cancer Science and Policy, National Cancer Center, Goyang, Korea

Acknowledgments / Grants

본 연구는 국립암센터 기관고유 과제(#1610402)의 지원을 받아 수행되었습니다.

*** Corresponding Author:**

Jae Kwan Jun, MD, PhD
National Cancer Control Institute, National Cancer Center, Goyang, Korea

Address: 323 Ilsan-ro, Ilsandong-gu, Goyang 10408, Korea
Phone: 82-31-920-2184 Fax: 82-31-920-2929
Email: jkjun@ncc.re.kr

(Running title: Survey About Lung Cancer Screening and Radiation Exposure)

Volume: 40, Article ID: e2018002

https://doi.org/10.4178/epih.e2018002

초록(Abstract)

목적(Purpose): 본 연구에서는 국내 폐암검진 및 그에 따르는 의료방사선 노출에 대한 인식 조사를 통해, 실제 의료기관에서의 현황과 함께 검진 관련 각종 인지도를 평가함으로써 향후 개선 혹은 보완점을 제시하고자 하였다.
방법(Methods): 전국 국가암검진 기관 중 층화 무작위 표본추출을 통해 선택된 검진기관의 전문의들을 대상으로 구조화된 설문지를 이용한 일대일 면접조사를 실시하였다. 검사 도구에 따른 폐암검진의 효과에 대한 인식과 더불어 폐암검진 대상의 선정과 권유 기준, 검사 장비의 종류, 검진에 따르는 부작용의 심각성에 대한 인식을 조사하였으며, 검진으로 인한 방사선 노출 위험을 과소평가할 오즈비를 로지스틱 회귀분석을 통해 산출하였다.
결과(Results): 흉부 엑스레이가 폐암검진에 효과적이라는 응답과 폐암검진 시행 여부의 결정 시에 흡연력을 고려하지 않는다는 응답이 각기 전체 응답자의 60%를 초과하여, 불필요한 검사의 시행 가능성을 시사하였다. 폐암검진의 부작용 중 위양성 및 방사선노출, 과진단에 대해서 응답자의 85% 내외가 무시해도 좋다는 답을 하였고, 70% 내외의 응답자들이 폐암검진에 따르는 방사선 피폭량을 실제보다 과소평가하였으며, 환자들에게 방사선 노출의 위험성을 교육하는 의사들의 비율 역시 낮았다.
결론(Conclusions): 대다수의 의사들이 방사선 노출을 비롯한 폐암검진의 위해를 과소평가하고 있음과, 폐암검진의 올바른 검사 도구에 대한 인식이 부족함을 확인할 수 있었다. 검진 프로그램의 성공적인 수행을 위해서는 검진 권고안 및 수반되는 부작용에 대한 의사들의 정확한 인지가 필요하다는 것을 기억해야 하겠다.

주제어(Keywords)

Lung Neoplasms; Early Detection of Cancer; Radiography; Computed tomography; Radiation Exposure; Health Care Surveys

Volume: 40, Article ID: e2018002

https://doi.org/10.4178/epih.e2018002

서론(Introduction)

저선량 흉부 컴퓨터 단층촬영(Low-dose Computed Tomography, LDCT)을 이용한 폐암 고위험군 대상 정기검진의 폐암 사망률 감소 효과를 보고한 미국 임상시험(National Lung Screening Trial, NLST)의 결과 보고 [1] 이후로, 여러 단체들에서 이러한 프로토콜에 따르는 폐암 조기검진 권고안 또는 개정안이 발표되었다 [2-6]. 뒤이어 이러한 권고안에 대한 의사들의 인지도와 더불어 검진 수행에 있어서의 장해요인이나 우려사항 등을 검진 수행 현황과 함께 조사한 다양한 연구들이 진행되었다 [7-13]. 이를 통해 대상자에게 검사를 권고하고 처방을 내리는 의사가 가지는 검진에 대한 인식 또는 태도는 실제 폐암검진의 시행 양상에 중요한 영향을 미치며, 결과적으로 제도의 성공적인 정착 여부를 결정짓는 요인이 됨을 알게 되었다 [14].

한국에서도 2015년에 LDCT를 이용한 폐암검진 권고안이 새롭게 발표된 바 있으며 [15], 현재 해당 검사의 국가암검진 도입을 검토하기 위한 시범사업이 실시 중에 있다. 이에 따라 의료현장에서 근무하는 일차진료의 및 전문의들의 폐암검진에 대한 올바른 인식과 태도가 제도의 시행 및 정착을 위한 선결 과제로 요구되는 실정이다. 특별히 LDCT는 흉부 엑스레이나 검진용 유방촬영검사 등 다른 방사선 검사에 비해서도 피폭 유효선량이 5~10배 가량 높기에 [16, 17] 검진의 이득과 위해를 평가함에 있어 방사선 노출에 대한 고려가 필수적이며, 이에 대한 적절한 검토와 관리가 제도의 도입 시 중요한 부분이라 할 수 있다.

그럼에도 불구하고 국내에서는 폐암검진 가이드라인의 변화 이후, 실제 의료 현장에서의 검진 실태 혹은 의료인들의 인식 조사 결과 등은 아직 보고된 바가 없다. 이에 본 연구에서는 전국 국가암검진 기관에 근무하는 전문의들의 표본집단을 대상으로 폐암검진 및 그로 인한 의료방사선 노출에 대한 인식 조사를 시행하여, 실제 검진이 어떤 수단을 통해 어떻게 이루어지고 있으며 이에 대한 의사들의 태도는 어떠한지를 응답을 통해 평가하였다. 이로써 각 의료기관에서의 폐암검진 관련 현황을 살펴보고, 의료진의 인식이 부족하여 개선 또는 보완이 필요한 영역들에 대한 근거를 제시하고자 한다.

연구대상 및 방법(Materials and Methods)

국가암검진 사업에 참여하는 전국 5천여 개의 검진기관을 대상으로 지역 및 검진기관 종별로 층화 무작위 표본추출을 시행하여 150개의 조사대상 기관을 선정하였다. 이 중 유선

Volume: 40, Article ID: e2018002

https://doi.org/10.4178/epih.e2018002

연락을 통해 설문 참여 동의를 구득한 104개 기관을 직접 방문하여, 소속 전문의 각 1명씩을 대상으로 일대일 면접조사를 통해 폐암검진에 대한 인식 조사를 실시하였다. 본 조사는 전문 조사기관에 의해 2013년 1월부터 2월까지 진행되었으며, 구조화된 설문지를 이용하여 폐암검진 및 의료 방사선 노출에 대한 인식 조사, 폐암검진 실시 현황 및 응답자 개인과 기관 특성에 대한 조사가 시행되었다.

폐암검진과 의료 방사선 노출에 대한 인식도 조사에서는 전체 응답자를 대상으로 검사도구에 따른 폐암검진의 효과에 대한 인식 및 LDCT 검진에 대한 인지도, 폐암검진 시 방사선 노출 위험과 관련한 인식을 측정하였다. 폐암검진의 효과에 대한 인식은 각 흡연력 및 검사방법 별로 “성인이 흉부 엑스레이/LDCT를 정기적으로 받는 것이 폐암 사망률 감소에 효과적이라고 생각하십니까?” 라는 질문을 통해, 인지하는 폐암검진의 방사선 노출 위험에 대한 대응으로서의 행동은 “흉부 엑스레이/LDCT로 폐암검진을 실시할 경우 방사선 위험에 대해 환자에게 교육하는 비율은 어느 정도입니까?” 라는 문항을 통해 평가하였다. 한편 폐암검진 실시 현황에 대한 설문은 “귀하는 혹은 귀하가 종사하는 의료기관은 폐암검진을 실시하고 있습니까?” 라는 질문에 대해 ‘실시하고 있다’ 라고 답한 응답자만을 대상으로 이루어졌는데, 실제 검진에서 “대상자의 흡연력 및 연령을 고려하여 검진여부를 결정하는지” 와 “무슨 검사를 실시하는지”에 대해 질문하였고, “폐암검진 시행 시 발생할 수 있는 잠재적인 부작용(위양성, 위음성, 방사선 노출, 과진단)에 대해 얼마나 심각하다고 생각하십니까?” 라는 문항을 통해 폐암검진의 위해와 그 심각성에 대한 인식을 측정하였다.

각 설문 항목에 대한 빈도와 백분율을 산출하여 기술하였으며, 폐암검진 시 방사선 노출 위험에 대한 인식도를 측정하는 문항인 “흉부 엑스레이/LDCT 검사 시 노출되는 의료 방사선 총량이 건강에 어느 정도 위험하다고 생각하십니까?”, “폐암검진 시행 시 방사선 노출의 부작용 발생 가능성에 대해 얼마나 심각하다고 생각하십니까?”, “흉부 엑스레이/LDCT 검사 1회 촬영 시 노출되는 방사선량이 어느 정도 된다고 생각하십니까?” 라는 5개 질문에 대하여 각각 ‘전혀 위험하지 않다’, ‘전혀 고려하지 않는다(무시한다)’ 라는 응답 및 실제보다 낮게 흉부 엑스레이 또는 LDCT 촬영시의 노출 방사선량에 대해 응답한 경우를 각기 집계함으로써 폐암검진으로 인한 방사선 노출 위험을 과소평가할 위험도를 로지스틱 회귀분석을 이용하여 산출하였다.

모든 설문 결과는 취합되어 조사 기간 종료 후에 분석되었고, 응답자의 개인 정보는 보호되었다. 본 연구는 국립암센터 기관심의위원회의 승인을 받아 진행되었으며(IRB No.:

NCCNC2015-0095), 통계적 분석은 SAS Software version 9.4 (SAS Institute Inc., Cary, NC) 패키지를 사용하였다.

연구결과(Results)

설문 응답자의 특성

목표 조사 대상이었던 150개 검진기관 중에서 총 104개 기관, 곧 104명의 전문의가 설문에 응답하여 약 69.3%의 참여율을 기록하였다. 이들 중 폐암검진을 실시한다고 응답한 의료기관은 54개(51.9%)였으며, 이러한 기관의 응답자들 중에서는 내과전문의가 37명(68.5%), 가정의학과 전문의가 8명(14.8%)이었다. 전체 응답자의 44.2%가 의사면허 취득 후 20년 미만 경과한 대상자였고, 나머지 55.8%는 20-40년이 경과한 것으로 응답하였다. 근무하는 의료기관은 대부분이 의원급 의료기관이었으며(99명, 95.2%), 병원급(4명, 3.8%)과 종합병원급 의료기관(1명, 1.0%)이 일부 존재하였다(표 1).

폐암검진의 도구와 타당성에 대한 인식 및 현황

흉부 엑스레이를 이용한 정기적 검진과 LDCT를 이용한 정기적 검진 모두에서 폐암 사망률 감소에 대해 '효과적'('매우 효과적'+'비교적 효과적')이라는 응답이 '비효과적'('전혀 효과적이지 않음'+'효과적이지 않음')이라는 응답에 비해 높았으며, 흡연력이 높을수록 '효과적'이라는 의견이 높게 나타났다. LDCT를 이용한 폐암검진의 효과를 보고한 미국의 NLST 연구결과에 대해서는 전체 응답자의 17.3%가 인지하고 있다고 답하였다(표 2).

응답자가 폐암검진을 실시하고 있다고 답한 경우, "폐암검진으로 무슨 검사를 실시하고 있습니까?" 라는 복수응답 질문에 대해서는 96.3%에서 흉부 엑스레이만을 이용하여 폐암검진을 실시하고 있다고 밝혔으며, LDCT를 이용한 검진을 수행하는 기관은 응답 실시기관의 18.5%였다. 실제 검진 여부 결정 시에는 흡연력을 고려하지 않는다는 응답이 66.7%로 고려한다는 응답보다 더 높았던 반면, 연령은 고려한다는 응답이 53.7%로 더 높았다.

폐암검진의 위해와 심각성에 대한 인식

폐암검진 시행 시 발생할 수 있는 잠재적인 부작용에 대하여, 위음성에 대해 '심각하다'('매우 심각하다'+'심각하다')고 응답한 비율이 53.7%로 가장 높았고, 위양성 및 방사선 노출, 과진단에 대해 '심각하다'고 응답한 비율은 각기 13.0%, 16.7%, 20.4%로 비교적 낮게 나타났다(그림 1). 흉부 엑스레이, LDCT를 실시할 경우 각각 방사선 위험에 대해 환자에게 교육하는 비율이 대략 몇 % 정도인지를 묻는 질문에서는 '전혀 교육하지 않는다(0%)'

는 응답이 흉부 엑스레이의 경우 전체의 74.0%로 약 4분의 3을 차지하였고, LDCT의 경우에는 응답자 중 33.7%로 3분의 1 이상을 차지하였다(그림 2).

폐암검진으로 인한 방사선 노출에 대한 인식

LDCT 검사 시 노출되는 의료방사선 총량의 건강 유해 정도에 대해 전체 응답자의 6.7%가 '전혀 위험하지 않다'고 답했으며, LDCT 촬영 1회 당 노출되는 유효방사선량('0.009 mSv 이하', '0.01-0.9 mSv 이하', '1.0-4.9 mSv 이하', '5.0-9.9 mSv 이하')에 대해서는 전체의 28.8% 만이 정답지인 '1.0-4.9 mSv 이하'를 선택하였고 대다수인 67.3%는 이보다 낮은 유효선량을 선택하였다. 흉부 엑스레이 검사 시 노출되는 의료방사선 총량의 건강 유해 정도와 촬영 1회 당 유효방사선량을 묻는 질문(답가지는 LDCT와 동일)에 대해서는 각각 53.9%, 73.1%의 응답자들이 '전혀 위험하지 않다'는 응답 및 실제 노출량(약 0.02 mSv)보다 낮은 값의 답가지를 선택하였다. 폐암검진 시 방사선노출 부작용에 대한 심각성 인식 문항에서는 폐암검진을 시행하고 있다고 응답한 응답자의 5.6%가 '전혀 고려하지 않는다(무시한다)'는 응답을 선택하였다. 위 다섯 개 질문에 대한 답변 결과를 토대로 응답자 및 기관의 특성에 따라 폐암검진으로 인한 방사선 노출 위험을 과소평가할 위험도를 산출한 결과, 타 전공에 비해 내과 혹은 가정의학과 전문의 집단에서 통계적으로 유의하게 낮은 오즈비를 나타내었고(adjusted OR=0.64, 95% CI: 0.43 to 0.96), 통계적으로 유의하지는 않았으나 여성 및 의사면허 취득 후 20년 미만 경과한 집단에서 그렇지 않은 집단에 비해 낮은 위험도가 관찰되었다 (표 3).

고찰(Discussion)

2011년 미국 NLST 연구의 결과 보고 이후, 국내외적으로 폐암검진이나 예방 혹은 관리 관련 프로토콜들이 많은 변화를 거치면서 현재까지도 계속적인 논의 중에 있다. 하지만 표본추출을 거친 전국 국가암검진기관의 전문의들을 대상으로 수행한 본 설문조사 연구를 통해, 우리는 실제 진료 현장에서 폐암검진과 관련한 의료진들의 인식 개선이 필히 선결되어야 할 중요한 과제임을 알 수 있었다. 연구 결과 진단검사 장비를 이용한 폐암 조기검진 시행의 효과 및 위해와 관련하여 잘못 인지하고 있는 의사들의 비율이 높았으며, 특히 방사선 노출에 있어서는 상당수의 응답자들이 그 위험성을 실제보다 과소평가하고 있었다.

효과적인 폐암검진 방법을 찾기 위한 연구는 1900년대 중반부터 미국과 유럽을 중심으로 꾸준히 행해져 왔다. 초반의 연구들은 주로 흉부 엑스레이를 이용한 검사에 대한 것이었으

며, 이들을 통해 얻어진 최종 결론은 잦은 또는 정기적인 흉부 엑스레이 검사는 고위험군 여부와 관계없이 폐암 사망률을 효과적으로 감소시키지 못한다는 것이었다 [18]. 따라서 우리나라의 가이드라인을 포함하여 대부분의 권고안에서는 흉부 엑스레이를 통한 폐암검진은 시행하지 말 것을 강조하고 있다 [3, 4, 15]. 하지만 본 연구를 통해, 아직도 우리나라에서는 상당수의 의사들이 흉부 엑스레이를 폐암 사망률 감소에 효과적인 검진 도구로 인식하고 있음과, 실제로도 대다수의 의료기관에서 엑스레이 검사를 이용하여 폐암검진이 이루어지고 있다는 것을 확인할 수 있었다. 이는 비효과적인 검사의 시행으로 인한 불필요한 비용 및 방사선 노출의 발생이라는 점에서 중요한 문제점으로 꼽을 수 있는 사항이었다.

반면 2000년을 전후하여 시작된 LDCT를 이용한 폐암검진에 관한 연구들에서는 대부분 LDCT가 폐암의 조기발견에 우수한 성과를 보인다는 결론을 얻을 수 있었으며, 가장 높은 근거수준을 지닌 것으로 평가된 NLST 연구에서는 사망률 감소 효과까지도 보고가 되었다 [5, 18]. 하지만 그럼에도 LDCT는 동반되는 위해의 불확실성으로 인해 검진의 적용 대상은 극히 제한적이다. 높은 양성률 및 그로 인한 불필요한 추가검사 혹은 중재시술, 이에 따르는 합병증의 위험, 촬영으로 인한 방사선 피폭, 과진단 등이 주로 논의되는 위해인데 [3-5, 15], 설령 이로 인한 피해가 크지 않다 하더라도 이것이 곧 이러한 부작용들을 무시해도 좋다는 것을 의미하는 것은 아니다. 특히 정기적 검사로 인한 방사선 노출의 위해는 아직 연구가 더 필요한 영역이며, 현재 시점에서 이러한 방사선 피폭이나 추가 검사 과정에 따르는 위험이 조기 암 발견이나 예측되는 사망률 감소에서의 이득을 위해 감수할 만한 정도의 것인지에 대한 결론을 내리기는 어려운 상황이다 [5, 19]. 따라서 전문가들은 LDCT를 이용한 폐암검진 시, 양성 판정 혹은 결절 발견 이후 검사에 대한 적절한 프로토콜과 함께 검진 과정에서의 방사선 피폭을 최소화고 이를 관리하는 기준이 반드시 있어야 함을 강조한다 [4, 5, 20].

2004년에 Brenner는 50-75세의 성인 흡연자를 대상으로 LDCT를 이용한 검진을 매년 시행할 경우 방사선 노출로 인해 발생하는 추가적인 폐암의 위험도와, 이를 미국의 전체 대상자에게 시행했을 때 예상되는 추가 폐암 환자수를 계산하여 보고하였다 [21]. Mccunney & Li는 2014년 발표한 논문에서, NLST 연구에서의 추적관찰 프로토콜에 따라 LDCT 검진에서 발견된 폐 결절을 회 당 8 mSv 선량의 흉부 CT로 추적관찰을 시행할 경우 20년 또는 30년 간 누적 피폭선량을 보고하였으며 [22], 미국 의학회지에 발표된 폐암 LDCT 검진의 이득과 위해에 관한 논문에서는 원폭 생존자 또는 의료방사선 연구에서의 방

Volume: 40, Article ID: e2018002

https://doi.org/10.4178/epih.e2018002

사선 노출량 모델을 이용하여 NLST 연구에서의 방사선 노출에 의한 암 사망을 예측하였다 [5]. 위 연구 결과들을 통해, 우리는 비록 검진으로 인한 방사선 피폭이 이득에 비해 감당할 만한 것이라는 주장을 감안하더라도 이것이 개인 차원을 넘어 '검진 프로그램' 즉 인구집단 수준의 집단적 노출이 된다면 공중보건학적으로 반드시 고려해야 할 중요한 문제가 된다는 것을 인지할 필요가 있다 [16, 22]. 한편 이와 같은 방사선 노출로 인한 개인별 위해의 크기는 수검 대상자의 연령이나 성별, 흡연력, 비만도, 그간의 방사선 노출력 및 검진 주기나 프로토콜 등에 따라 크게 달라질 수 있으므로 [20, 23], 이를 고려하여 권고 여부와 그 대상이 달라져야 하고 그에 따른 적절한 검사 지침을 제시 및 배포함으로써 의료진들의 인식도를 보다 높일 필요가 있다. 특별히 흡연과 방사선 노출은 잠재적인 상승효과의 가능성까지도 있는 것으로 보여져 더욱 주의가 필요한 부분이라 하겠다 [16, 22].

본 설문조사에서 이러한 방사선 노출의 위해에 대하여 실제보다 과소평가 혹은 간과할 위험도를 응답자의 특성에 따라 산출해 본 결과, 전문의 전공 과목에 따라서 통계적으로 유의한 차이를 관찰할 수 있었다. 타 전공에 비해 내과 또는 가정의학과 전문의의 경우 그러한 위험성이 유의하게 낮았는데, 이와 같은 결과는 조사 대상이었던 일차진료의 집단에서 전공에 따라 검진에 대한 올바른 접근이나 이해의 정도가 달라질 수 있음을 보여주었다. 즉 내과나 가정의학과와 같은 수련 과정을 통해 1차, 2차 예방 및 검진 등 보다 넓은 의료의 개념에 대해 익힌 경우에는 그에 수반되는 위해 또는 부작용에 대해서도 바르게 인식할 수 있으나, 그렇지 않은 경우에는 수련 과정 혹은 이후의 교육 등을 통한 지속적 평가와 관리로써 취약한 부분을 보완할 필요성이 있음을 보여 준 것이다. 한편 많은 수의 응답자들이 폐암검진의 위음성에 대한 심각성을 높이 평가하였는데 여기에는 대다수가 검진 수단으로 흉부 엑스레이를 고려한 영향도 있었을 것으로 보이며, 반면에 LDCT를 이용한 검진의 주요 부작용인 위양성 및 방사선 피폭, 과진단에 대해서는 무시할 수 있다는 응답이 80% 전후로 높게 나타나 검사의 위해에 대한 인식도가 낮음을 보여주었다. 또한 다수의 응답자들이 LDCT 촬영에 따른 방사선 피폭량 역시 과소평가하고 있었는데, LDCT 1회 촬영시의 방사선 노출 유효선량은 평균적으로 약 1.5-2.0 mSv 정도인 것으로 보고되는 데 비해 [5, 22, 24] 전체 응답자의 약 67.3%가 이를 과소평가하고 있었다. 특히 CT 촬영에 수반되는 방사선 피폭량은 장비의 종류나 촬영 설정에 따라 변동의 폭이 넓을 수 있으며, 매년 검진용 검사에 더하여 필요시의 진단용 검사까지 행해지다 보면 총 유효선량은 더욱 높아질 수 있기에 정확한 평가와 인지가 더욱 요구된다.

이렇듯 검진 권고안에 대한 올바른 숙지는 현재 유일하게 효과적인 검진 도구로 인정되고 있는 LDCT를 이용한 검진이 필요한 대상자들에게 적절히 시행되도록 할 뿐 아니라, 불필요한 검진으로 인한 위해 발생을 최소화하는 데 있어서도 필수적이다. 본 연구는 폐암검진에 대한 의료제공자의 인식 및 검진 현황을 설문을 통해 평가함으로써, 제도의 도입 이전에 보완해야 할 부족한 부분들을 검토하고 개선 방안을 제시하는 근거 자료를 제공하였다는 의의를 가진다. 하지만 조사 시행 시기가 2013년 1-2월로, 외국의 주요 권고안들이 발표된 시기인 2012-2015년에 비추어 보아도 초반부일 뿐 아니라 국내 가이드라인이 발표된 2015년 보다는 더욱 앞선 시기이기에 전반적으로 LDCT를 이용한 폐암검진에 대한 인지도가 상대적으로 낮을 수 밖에 없었을 것이라는 한계점을 지니며, 지금 시점에서 동일한 연구를 수행한다면 여러 측면에서 그 인지도가 향상되어 나타날 것으로 예측된다. 또한 조사에 참여한 기관의 분포 및 특성에도 제한점이 있었는데, 대부분의 검진기관이 1차 진료기관인 의원급 기관이어서 병원이나 종합병원급 의료기관의 현황은 알기가 어려웠다. 그리고 폐암검진을 시행한다고 응답한 참여 기관 중의 검진용 LDCT 장비 보급률이 매우 낮아 이를 이용한 폐암검진 수행률도 낮을 수 밖에 없었는데, 따라서 설문 문항 중 “폐암검진 시 시행하는 검사방법”에 대한 답변 결과는, 응답자의 문제라기보다는 의료기관의 한계로 인한 제한점이 작용했을 가능성이 크다고 할 수 있겠다. 그렇기에 향후에는 보다 다양한 의료기관을 대상으로 최근의 폐암검진 관련 현황을 파악하고, 우리나라의 실정에 맞게 제도를 도입 및 정착시킬 수 있는 방안에 대한 연구들이 계속해서 이루어져야 하겠다.

우리 연구는 처음으로 국내에서 수행된 폐암검진에 대한 의료인들의 인식 평가 결과 보고로서, 조사 결과 대다수의 의사들이 방사선 노출을 비롯한 폐암검진의 위해에 대해서는 과소평가하는 경향을 보였으며 효과적인 검진 수단과 대상에 대한 인식도 적절하지 못했다. 개인 및 인구집단을 대상으로 시행하는 검진의 이득은 항상 그에 따르는 위해와 함께 고려되어야 하기에, 각 의료 제공자는 LDCT를 이용한 폐암검진의 이득과 위해를 각기 바르게 이해하며, 검진 권고 대상자나 검사 방법의 선택에 있어서 가이드라인에 대한 정확한 인지를 바탕으로 판단을 내릴 수 있어야 하겠다. 향후 검진 대상 범위에 따른 이득과 위해 사이의 균형, 보험 급여 및 비용-효과성, 적정 질 관리와 그에 따르는 프로토콜의 확립 등에 대한 지속적 논의가 더 필요하며, 이와 더불어 일차진료의 및 전문의에 대한 교육 강화, 의사와 환자 간 의사결정 과정에 대한 적절한 지침 제시 등이 요구되는 과제라 할 수 있겠다.

Volume: 40, Article ID: e2018002

https://doi.org/10.4178/epih.e2018002

이해상충(Conflicts of interest)

저자들은 본 연구와 관련하여 어떠한 이해상충도 가지고 있지 않습니다.

Volume: 40, Article ID: e2018002

https://doi.org/10.4178/epih.e2018002

References

1. National Lung Screening Trial Research Team. Reduced lung-cancer mortality with low-dose computed tomographic screening. N Engl J Med 2011;2011:395-409.

2. Wood DE, Kazerooni E, Baum SL, Dransfield MT, Eapen GA, Ettinger DS, et al. Lung cancer screening, version 1.2015: featured updates to the NCCN guidelines. J Natl Compr Canc Netw 2015;13:23-33.

3. Moyer VA. Screening for lung cancer: US Preventive Services Task Force recommendation statement. Ann Intern Med 2014;160:330-338.

4. Wender R, Fontham ET, Barrera E, Colditz GA, Church TR, Ettinger DS, et al. American Cancer Society lung cancer screening guidelines. CA: a cancer journal for clinicians 2013;63:106-117.

5. Bach PB, Mirkin JN, Oliver TK, Azzoli CG, Berry DA, Brawley OW, et al. Benefits and harms of CT screening for lung cancer: a systematic review. JAMA 2012;307:2418-2429.

6. Jaklitsch MT, Jacobson FL, Austin JH, Field JK, Jett JR, Keshavjee S, et al. The American Association for Thoracic Surgery guidelines for lung cancer screening using low-dose computed tomography scans for lung cancer survivors and other high-risk groups. J Thorac Cardiovasc Surg 2012;144:33-38.

7. Raz DJ, Wu GX, Consunji M, Nelson RA, Kim H, Sun CL, et al. The Effect of Primary Care Physician Knowledge of Lung Cancer Screening Guidelines on Perceptions and Utilization of Low-Dose Computed Tomography. Clin Lung Cancer 2017.

8. Dunn J, Garvey G, Valery PC, Ball D, Fong KM, Vinod S, et al. Barriers to lung cancer care: health professionals' perspectives. Support Care Cancer 2017;25:497-504.

9. Hoffman RM, Sussman AL, Getrich CM, Rhyne RL, Crowell RE, Taylor KL, et al. Attitudes and Beliefs of Primary Care Providers in New Mexico About Lung Cancer Screening Using Low-Dose Computed Tomography. Prev Chronic Dis 2015;12:E108.

10. Iaccarino JM, Clark J, Bolton R, Kinsinger L, Kelley M, Slatore CG, et al. A National Survey of

Pulmonologists' Views on Low-Dose Computed Tomography Screening for Lung Cancer. Ann Am Thorac Soc 2015;12:1667-1675.

11. Klabunde CN, Marcus PM, Han PK, Richards TB, Vernon SW, Yuan G, et al. Lung cancer screening practices of primary care physicians: results from a national survey. Ann Fam Med 2012;10:102-110.

12. Lewis JA, Petty WJ, Tooze JA, Miller DP, Chiles C, Miller AA, et al. Low-dose CT lung cancer screening practices and attitudes among primary care providers at an academic medical center. Cancer Epidemiol Biomarkers Prev 2015;24:664-670.

13. Rajupet S, Doshi D, Wisnivesky JP, Lin JJ. Attitudes About Lung Cancer Screening: Primary Care Providers Versus Specialists. Clin Lung Cancer 2017.

14. Wiener RS, Gould MK, Arenberg DA, Au DH, Fennig K, Lamb CR, et al. An official American Thoracic Society/American College of Chest Physicians policy statement: implementation of low-dose computed tomography lung cancer screening programs in clinical practice. Am J Respir Crit Care Med 2015;192:881-891.

15. Jang SH, Sheen S, Kim HY, Yim HW, Park BY, Kim JW, et al. The Korean guideline for lung cancer screening. Journal of the Korean Medical Association 2015;58:291-301 (Korean).

16. Albert JM. Radiation risk from CT: implications for cancer screening. AJR Am J Roentgenol 2013;201:W81-87.

17. Korea Institute of Nuclear Safety (KINS). Design and construction of a radiation dose database for Korean population. Daejeon: KINS; 2005. Report No.: KINS/HR-673 (Korean).

18. Manser R, Lethaby A, Irving LB, Stone C, Byrnes G, Abramson MJ, et al. Screening for lung cancer. Cochrane Database Syst Rev 2013:Cd001991.

19. Dajac J, Kamdar J, Moats A, Nguyen B. To Screen or not to Screen: Low Dose Computed Tomography in Comparison to Chest Radiography or Usual Care in Reducing Morbidity and Mortality from Lung Cancer. Cureus 2016;8:e589.

20. Kauczor HU, Bonomo L, Gaga M, Nackaerts K, Peled N, Prokop M, et al. ESR/ERS white paper on lung cancer screening. Eur Respir J 2015;46:28-39.

21. Brenner DJ. Radiation risks potentially associated with low-dose CT screening of adult smokers for lung cancer. Radiology 2004;231:440-445.

22. McCunney RJ, Li J. Radiation risks in lung cancer screening programs: a comparison with nuclear industry workers and atomic bomb survivors. Chest 2014;145:618-624.

23. de Koning HJ, Meza R, Plevritis SK, ten Haaf K, Munshi VN, Jeon J, et al. Benefits and harms of computed tomography lung cancer screening strategies: a comparative modeling study for the U.S. Preventive Services Task Force. Ann Intern Med 2014;160:311-320.

24. Larke FJ, Kruger RL, Cagnon CH, Flynn MJ, McNitt-Gray MM, Wu X, et al. Estimated radiation dose associated with low-dose chest CT of average-size participants in the National Lung Screening Trial. AJR Am J Roentgenol 2011;197:1165-1169.

Volume: 40, Article ID: e2018002

https://doi.org/10.4178/epih.e2018002

Table 1. Characteristics of Study Subjects

Characteristics	Total		Physicians of self-reported lung cancer screening institutions[1]	
	n	(%)	n	(%)
Number of the respondents	104	(100.00)	54	(51.92)
Sex				
Male	83	(79.81)	47	(87.04)
Female	21	(20.19)	7	(12.96)
Age (years)				
30-39	15	(14.42)	7	(12.96)
40-49	49	(47.12)	24	(44.44)
≥50	40	(38.46)	23	(42.59)
Specialty				
Internal medicine	54	(51.92)	37	(68.52)
Family medicine	12	(11.54)	8	(14.81)
Others	38	(36.54)	9	(16.67)
Years after medical license issuance				
<10	8	(7.69)	9	(9.26)
10-19	38	(36.54)	5	(37.04)
20-29	49	(47.12)	11	(46.30)
≥30	9	(8.65)	15	(7.41)
Hospital type				
Clinic	99	(95.19)	51	(94.44)
Hospital	4	(3.85)	2	(3.70)
General hospital	1	(0.96)	1	(1.85)
Underestimation of the radiation exposure risk from lung cancer screening[2]				
Total number of responses	469[3]	(100.00)	269[4]	(57.36)
Underestimate	212	(45.20)	102	(37.92)
No underestimate	257	(54.80)	167	(62.08)

LDCT, low-dose computed tomography

[1] Physicians who replied the institutions they belonged to were conducting lung cancer screening

[2] Composed of 5 questions and corresponding answers, from which following responses for each question were defined as ‘underestimation’: (1) Answered ‘Never risky’ to the statement of “How risky do you think the total medical radiation dose by chest X-ray is to health?”; (2) Answered ‘Never risky’ to the statement of “How risky do you think the total medical radiation dose by chest LDCT is to health?”; (3) Answered ‘Never considered (ignored)’ to the statement of “How serious do you think is the chance of adverse effects by radiation exposure during lung cancer screening?”, among the physicians of self-reported lung cancer screening institutions (n=54) and non-respondent (n=1) was excluded; (4) Answered ‘<0.01 mSv’ to the question asking “Estimated effective radiation dose of one shot chest X-ray examination”; (5) Answered ‘<0.01 mSv’ or ‘0.01-0.9 mSv’ to the question asking “Estimated effective radiation dose of one LDCT imaging”

[3] The number of respondents was 104 (physicians) for 4 questions and 53 (physicians) for the other 1

Volume: 40, Article ID: e2018002

https://doi.org/10.4178/epih.e2018002

question.

[4] The number of respondents was 54 (physicians) for 4 questions and 53 (physicians) for the other 1 question.

Volume: 40, Article ID: e2018002

https://doi.org/10.4178/epih.e2018002

Table 2. Physicians' Perceptions and Practices about Lung Cancer Screening

Questions	Yes		No	
	**n**	**(%)**	**n**	**(%)**
Among all respondents (n=104)				
Do you think chest X-ray screening is effective to lung cancer mortality reduction?				
In non-smokers	62	(59.62)	42	(40.38)
In past smokers	73	(70.19)	31	(29.81)
In current smokers	76	(73.08)	28	(26.92)
Do you think chest LDCT screening is effective to lung cancer mortality reduction?				
In non-smokers	59	(56.73)	45	(43.27)
In past smokers	86	(82.69)	18	(17.31)
In current smokers	91	(87.50)	13	(12.50)
Are you familiar with the results of the NLST study regarding LDCT lung cancer screening?				
	18	(17.31)	86	(82.69)
Among the respondents of self-reported lung cancer screening institutions [1] (n=54)				
Which screening tests do you order?				
Chest X-ray	52	(96.30)		
LDCT	10	(18.52)		
Sputum cytology	6	(11.11)		
Do you recommend lung cancer screening tests depending on smoking status?				
	18	(33.33)	36	(66.67)
Do you recommend lung cancer screening tests depending on age? [2]				
	29	(54.72)	24	(45.28)

Volume: 40, Article ID: e2018002

https://doi.org/10.4178/epih.e2018002

LDCT, low-dose computed tomography; NLST, National Lung Screening Trial

[1] Respondents who replied the institutions they belonged to were conducting lung cancer screening

[2] Non-respondent (n=1) was excluded.

Volume: 40, Article ID: e2018002

https://doi.org/10.4178/epih.e2018002

Table 3. Odds Ratios for Underestimating the Risk of Radiation Exposure of Lung Cancer Screening

**Characteristics**	Total number of responses (5 questions)[1]	Underestimate[2]		No underestimate		**Crude OR**		**Adjusted OR**	
		n	(%)	n	(%)	Estimates	(95% CI)	Estimates	(95% CI)
**Sex**									
Male	379	169	(44.59)	210	(55.41)	1.00		1.00	
Female	90	43	(47.78)	47	(52.22)	1.14	(0.72-1.80)	0.98	(0.60-1.60)
**Years in practice**									
< 20 years	209	91	(43.54)	118	(56.46)	0.89	(0.61-1.28)	0.89	(0.62-1.29)
≥ 20 years	260	121	(46.54)	139	(53.46)	1.00		1.00	
**Specialty**									
Internal or Family medicine	309	128	(41.42)	181	(58.58)	0.64	(0.44-0.94)	0.64	(0.43-0.96)
Others	160	84	(52.50)	76	(47.50)	1.00		1.00	
**Type of medical facility**									
Clinic	446	201	(45.07)	245	(54.93)	0.90	(0.39-2.07)	0.83	(0.38-2.07)
Hospital	23	11	(47.83)	12	(52.17)	1.00		1.00	

[1] The number of respondents was 104 (physicians) for 4 questions and 53 (physicians) for the other 1 question.
[2] Criteria for 'underestimation' in each five question are described in manuscript (materials and methods section) and the footnote (number 2) of Table 1.

Volume: 40, Article ID: e2018002

https://doi.org/10.4178/epih.e2018002

Figure Legends

Figure 1. Physicians' Perceptions to the Potential Adverse Effects of Lung Cancer Screening*

* Among the physicians who replied the institutions they belonged to were conducting lung cancer screening (n=54); Non-respondent (n=1) was excluded.

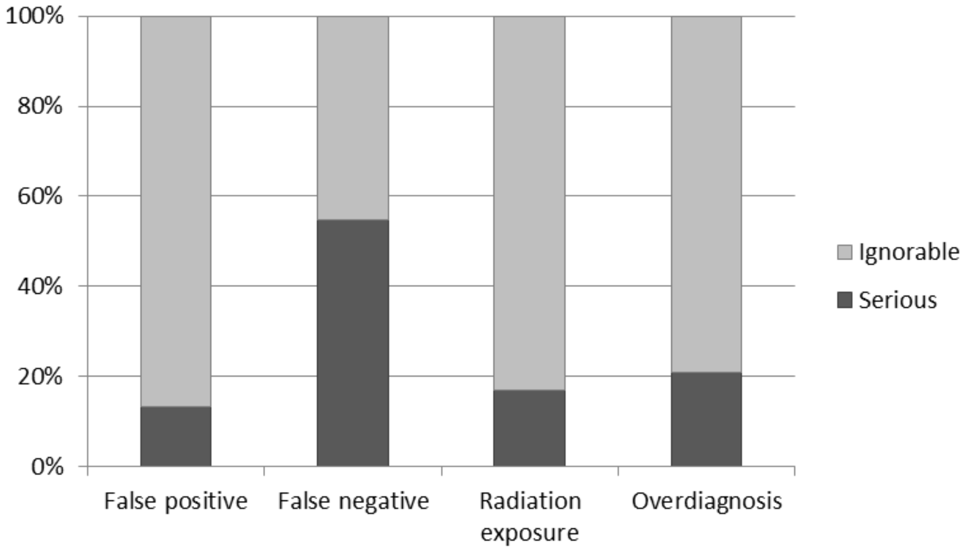

Volume: 40, Article ID: e2018002

https://doi.org/10.4178/epih.e2018002

Figure 2. Proportion of Providing Patient Education about Radiation Exposure before Lung Cancer Screening via Chest X-ray or LDCT*

(LDCT, low-dose computed tomography)

* Among the all respondents (n=104); For LDCT screening, non-respondents (n=4) were excluded.

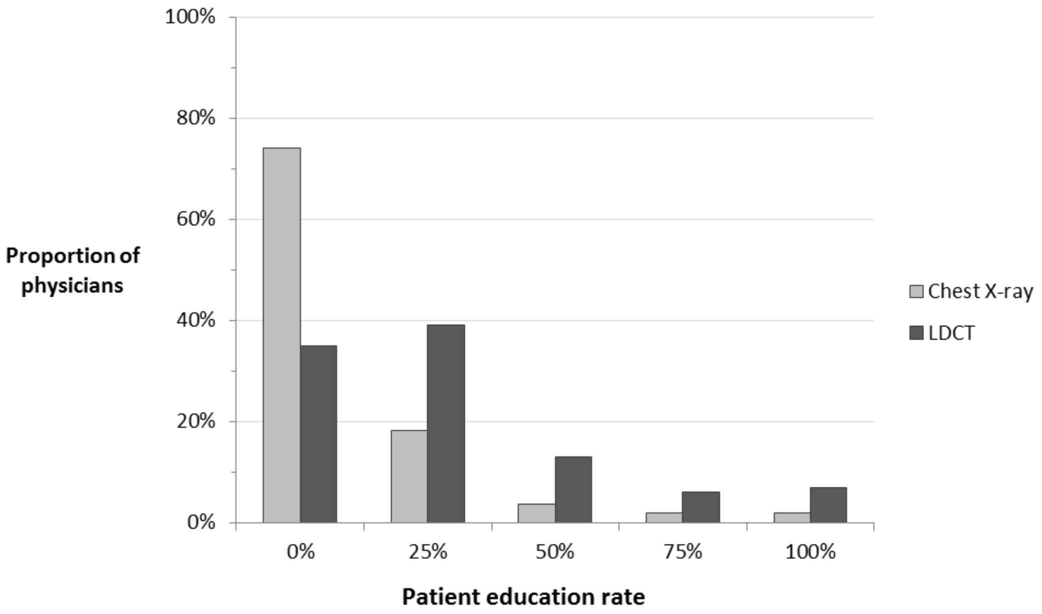

Supplement: Supplementary file 1 [file epih-40-e2018002-supplementary.pdf]
